# Flexor hallucis longus hypertrophy secondary to Achilles tendon tendinopathy: an MRI-based case–control study

**DOI:** 10.1007/s00590-021-02891-8

**Published:** 2021-02-08

**Authors:** Stephan H. Wirth, Octavian Andronic, Fabian Aregger, Anna Jungwirth-Weinberger, Thorsten Jentzsch, Andreas Hecker

**Affiliations:** Department of Orthopaedics, Balgrist University Hospital, University of Zurich, Forchstrasse 340, 8008 Zurich, Switzerland

**Keywords:** Achilles tendon tendinopathy, Flexor hallucis longus hypertrophy, Chronic achilles tendon rupture, Flexor hallucis longus transfer, Achilles tendon MRI

## Abstract

**Purpose:**

The purpose of this study was to outline an indirect sign of advanced Achilles tendinopathy on magnetic resonance imaging (MRI), based on the hypothesis that these patients would present with secondary hypertrophy of the flexor hallucis longus muscle (FHL).

**Methods:**

MRI scans of Achilles tendon were analyzed retrospectively in two cohorts. The study group consisted of consecutive patients presenting with clinical signs of Achilles tendinopathy and no previous surgeries, while the control group were patients that had an MRI due to other reasons and no signs of tendinopathy. Two parameters from two muscle bellies were measured and compared on axial MRI scans 4–5 cm above the ankle joint line at the level of greatest thickness: area and diameter of the triceps surae (TS) and of the FHL muscle. Ratios (FHL/TS) were calculated for area (Ar) and diameter (Dm) measurements. Interobserver agreement was analyzed. A receiver operating characteristic (ROC) curve was created for both ratios to assess potential cutoff points to differentiate between the groups.

**Results:**

A total of 60 patients for each study group were included. Both ratios Ar(FHL/TS) and Dm(FHL/TS) showed significant higher values in the tendinopathy group (*p* < 0.001). There were strong to very strong intraclass correlation coefficients (ICC = 0.75–0.93). A diameter ratio Dm (FHL/TS) of 2.0 or higher had a sensitivity of 49% and specificity of 90% for concomitant Achilles tendinopathy.

**Conclusion:**

In our patient cohort, FHL hypertrophy was observed in patients with Achilles tendinopathy as a possible compensatory mechanism. Measuring a diameter ratio Dm(FHL/TS) of 2.0 or higher on an axial MRI, may be indicative as an indirect sign of functional deterioration of the Achilles tendon.

## Background

Chronic Achilles tendinopathy represents a common disease that can affect professional athletes [1] and also people engaging occasionally in sports or even having a sedentary lifestyle [2]. In 2011, its incidence in a Dutch population was described as 2 per 1000 patients registered by a general practitioner (GP) [3]. In an histological study, 34% of the Achilles tendon specimens of spontaneously ruptured tendons were showing signs of tendinopathy, suggesting that the acute lesions also have a predisposing degeneration [4]. This further supports the importance of timely diagnosing and delivering the appropriate management.

First line of treatment is usually conservative and may take up months to demonstrate effectiveness. This includes reduction of load, stretching as well as eccentric muscle strengthening and may lead to a favorable outcome in 71% of the cases [5]. However, as many as 25% of patients fail conservative therapy and might benefit from surgery [6]. Surgical procedures, mostly consisting of debridement with direct repair, are considered when conservative treatment is not successful after an average period of 3–6 months [7, 8]. A variety of surgical options have been described without a definitive consensus on the best technique [9].

If there is advanced damage of the tendon [6], a tendon transfer is a recommended option [10–13]. One of the most widely used is the flexor hallucis longus (FHL) transfer [14]. Evidence demonstrated a hypertrophy of the FHL after performing a tendon transfer [15, 16]. Furthermore, recent biomechanical studies provide important evidence supporting FHL as transfer option, as FHL transfer significantly increases load to failure of Achilles tendons [17].

In our practice, there was a clinical observation that the FHL hypertrophy may even occur preoperatively in cases with severe Achilles tendinopathy as a compensatory mechanism. The purpose of this study was to outline this finding on magnetic resonance imaging (MRI), based on the hypothesis that patients with Achilles tendinopathy would present with secondary hypertrophy of the flexor hallucis longus muscle (FHL).

## Methods

Consecutive MRI scans between 01/2016 and 01/2017, that assessed the calf region and included the Achilles tendon, were extracted from the local institutional database. All patients that were included signed a written consent form. The study was approved by our institutional ethical review board and by the local Ethics Commission (BASEC Nr. 2018-00098). The study was carried out in accordance with the World Medical Association Declaration of Helsinki [18].

### Patient selection

Inclusion criteria for the tendinopathy group were patients that exhibited radiographic signs of Achilles tendon tendinopathy as defined by previous studies [19, 20], which included combinations of the following: intratendinous signal alterations, interstitial or insertional tears, peritendinous edema, loss of the physiological anterior concave margin of the tendon, irregular mucoid deposition, intratendinous multifocal speckled appearance with changes in volume. Only imaging of sufficient quality that included sagittal and axial views (T1 as well as T2 weighted/STIR (short tau inversion recovery)/with and without fat suppression) were included.

The control group included subjects without any documented complaints regarding the Achilles function and presented a normal radiographic morphology of the Achilles tendon on MRI. Exclusion criteria in both groups were relevant morphologic changes of the upper ankle joint, the hindfoot or the calf muscles as well as history of relevant previous surgery or injury of the lower limb.

### Radiographic measurement strategy

**Fig. 1:**
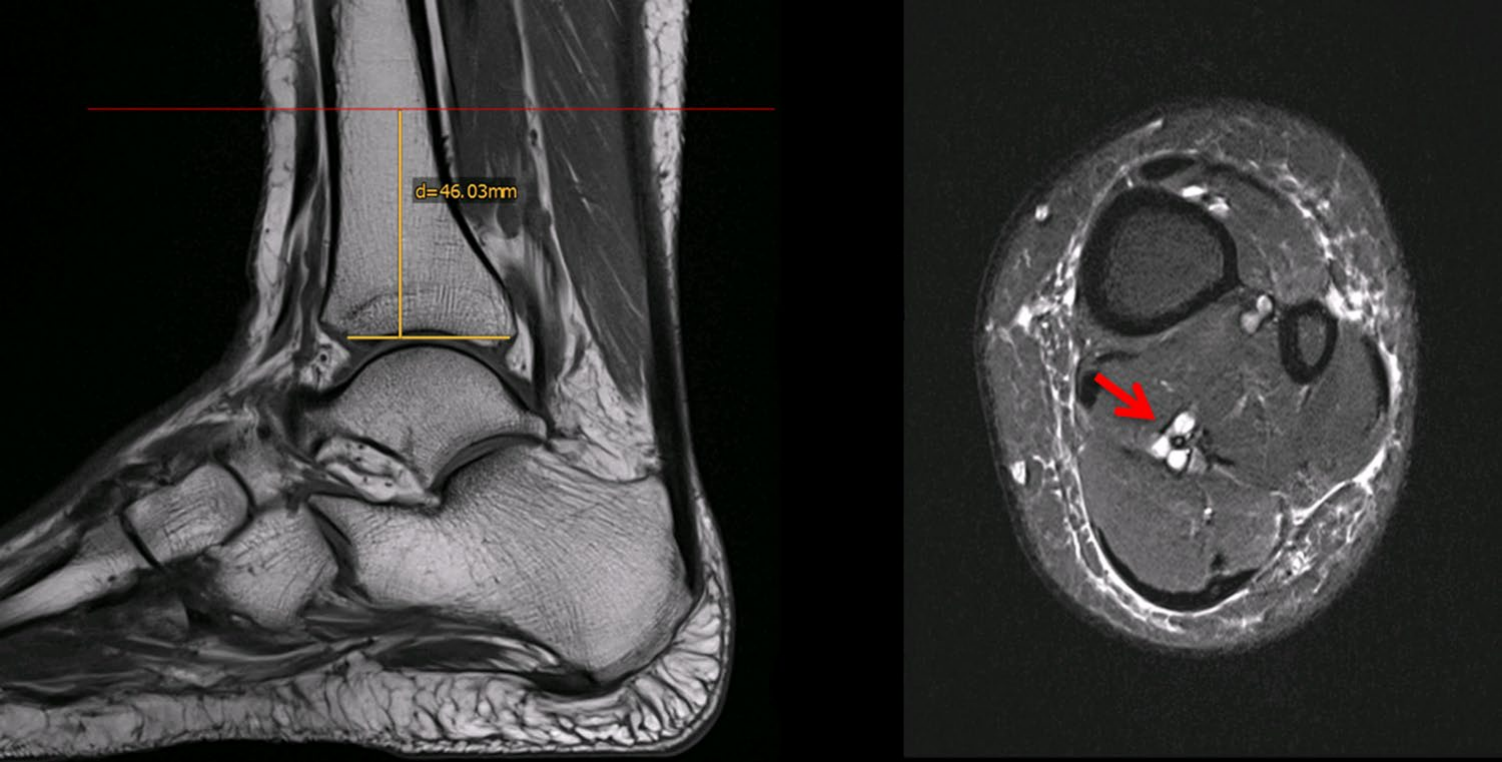
The height was determined on the sagittal plane by calculating the distance from the upper ankle joint line. The red arrow marks the neurovascular bundle (color figure online)

**Fig. 2:**
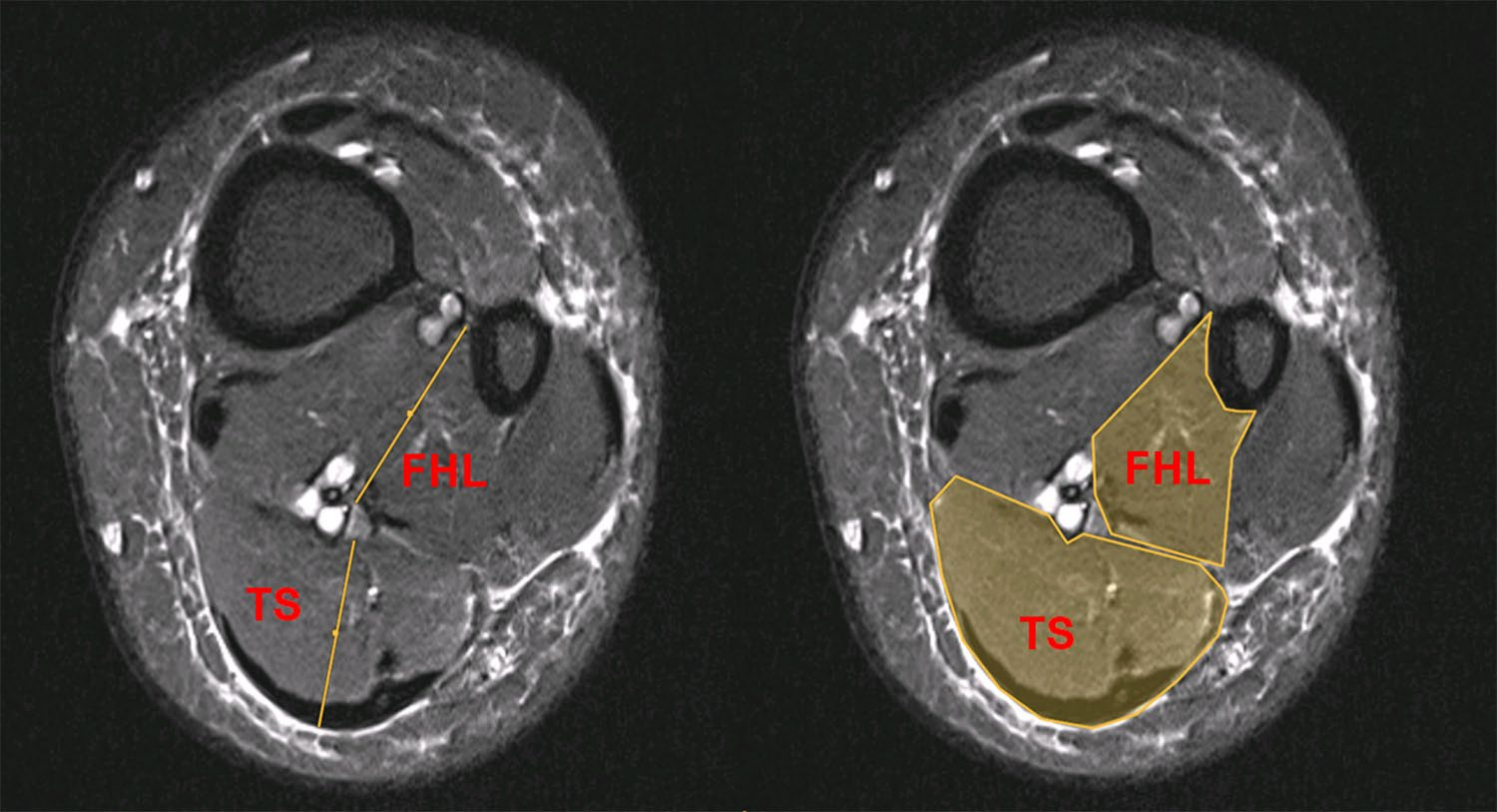
Measurement of diameter and area of FHL and TS muscle bellies

**Fig. 3:**
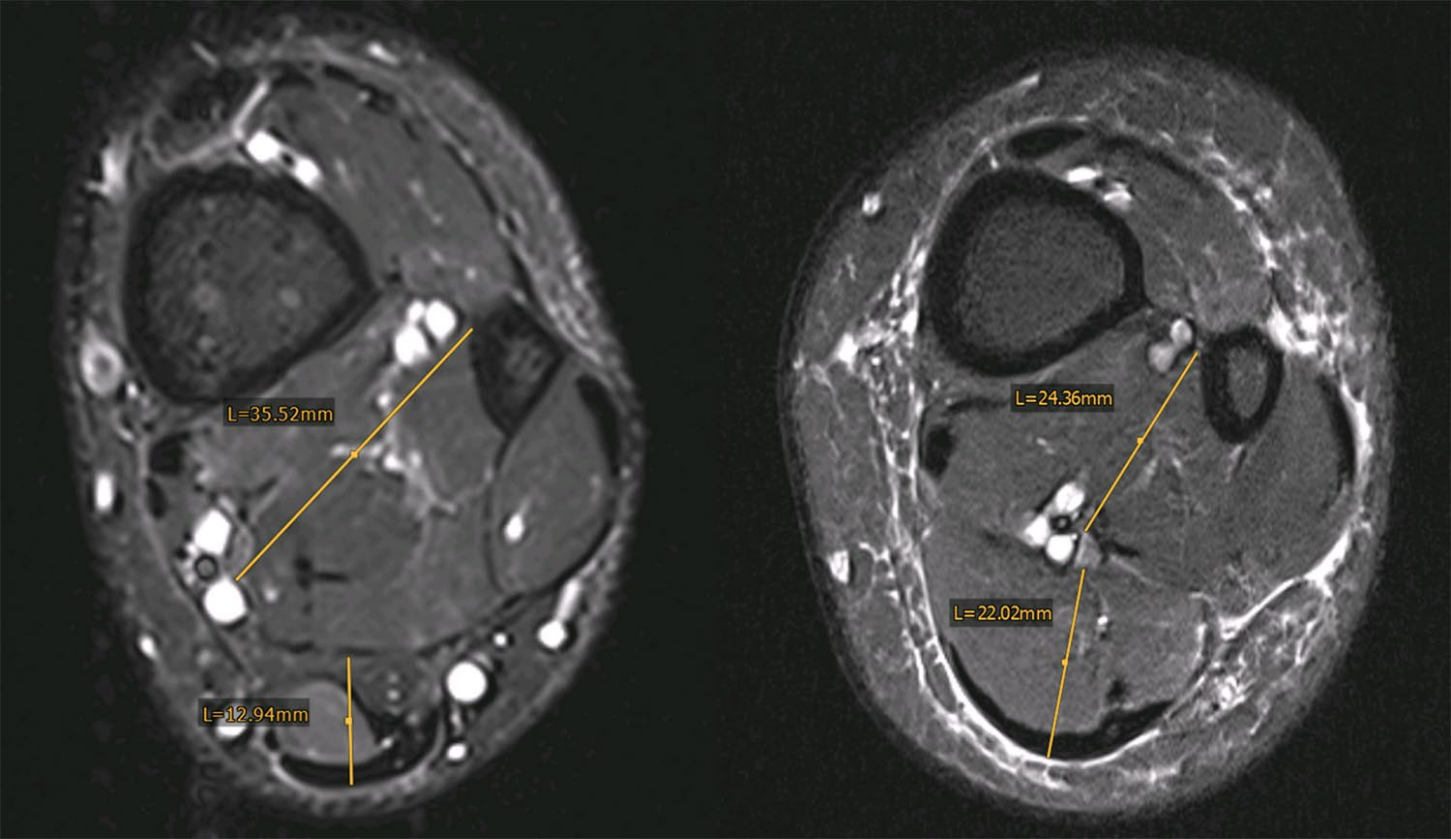
An example of two participants (left from Achilles tendinopathy group and on the right-side control group). Obvious quantitative differences can be observed

Axial MRI scans (slice thickness 6 mm) were analyzed on a level 4–5 cm above the ankle joint line at the level of greatest thickness for each muscle belly. This height was chosen as it was consistently representative of a good portion of muscle belly for both FHL and triceps surae/soleus. A sagittal view was then used to verify the height (Fig. 1). Next, for measurement of the diameter of the TS, a perpendicular line was drawn to the frontal plane through the midline of the muscle belly. The FHL diameter was measured as the distance from the medical corner of the fibula to the posteromedial corner of the FHL muscle (Fig. 2), identified as the location where the neurovascular bundle runs along the FHL. Measurements were made in both groups by two independent investigators (Fig. 3). Next, ratios of the area—Ar(FHL/TS) and of the diameter—Dm(FHL/TS) were calculated.

### Statistical analysis

Data were mainly non-normally distributed. Medians and interquartile ranges (IQR) are given. Spearman correlation analysis tested for interobserver agreement. The Wilcoxon rank sum test was used to compare measurements between groups. Intraclass correlation coefficients (ICC) for individual agreements with F tests were calculated for interrater agreement. We also provide minimal detectable change and standard error of measurement values. A logistic regression model was fitted to account for the potential confounder age, sex, and side. A receiver operating characteristic (ROC) curve was created for both quotients to assess potential cutoff points. A post-hoc power analysis using a test comparing two independent means yielded a sufficient power (power = 0.99 for the quotient of the diameter of FHL divided by TS using the mean and standard deviations (SD) for both groups (2.16 (SD 0.62) and 1.71 (SD 0.32) (n = 120)). Stata/IC (version 13.1; StataCorp LP, College Station, TX, USA) was used.

## Results

Demographics of the study participants are represented in Table 1. The mean height of measurement was 4.5 cm above the ankle joint in both groups.

**Table 1:** Demographics of continuous and categorical data (*n* = 120)

Variable	Tendinopathy (median [IQR])		
	Yes (*n* = 60)	No (*n* = 60)	*p*-Value
Age (y)	60 (16)	38 (31)	< 0.001*
Gender			
Females	24 (40%)	39 (65%)	0.006^†^
Males	36 (60%)	21 (35%)	
Side			
Right	34 (57%)	26 (43%)	0.144^†^
Left	26 (43%)	34 (57%)	

*IQR* interquartile range, *y* years

*Wilcoxon rank sum test

^†^Chi-squared test

The diameter ratio Dm (FHL/TS) showed significantly higher values in the tendinopathy group than the control group, *p* < 0.001 (median = 2.0 [IQR = 0.8] vs 1.7 [0.3]) (Table 2). Similar results were obtained by calculating the ratio regarding the area Ar (FHL/TS), which also showed significantly higher values in the tendinopathy group than in the control group (1.8 [1.3] vs 1.3 [0.7]), *p* < 0.001.

**Table 2:** Measurements comparing Achilles tendinopathy group to controls

Variable	Tendinopathy (median [IQR])				
	Yes (*n* = 60)	No (*n* = 60)	*p* value*	OR (95% CI)	*p* value^†^
Diameter (mm)					
FHL	29.5 (6.8)	26.5 (5.0)	< 0.001	1.24 (1.06–1.44)	0.006
TS	14.8 (4.5)	16.0 (3.5)	0.006	0.82 (0.70–0.96)	0.015
FHL/TS	2.0 (0.8)	1.7 (0.3)	< 0.001	9.56 (2.46–37.22)	0.001
Area (mm^2^)					
FHL	545.0 (122.3)	453.3 (131.5)	0.080	1.01 (1.00–1.01)	0.002
TS	274.5 (182.0)	331.0 (140.5)	< 0.001	1.00 (0.99–1.12)	0.236
FHL/TS	1.8 (1.3)	1.3 (0.7)	< 0.001	2.52 (1.33–4.78)	0.005

*IQR* interquartile range, *OR* odds ratio, *%* percent, *CI* confidence interval, *FHL* flexor hallucis longus, *TS* triceps surae

*Wilcoxon rank sum test

^†^Wald test in a logistic regression model adjusting for age, sex, and side

A strong to very strong ICC regarding all measurements was determined (0.75–0.93) (Table 3).

**Table 3:** Intraclass correlation coefficient

Variable	ICC (95% CI)	MDC	SEM	*p* value*
Diameter (mm)				
FHL	0.75 (0.66–0.82)	2.55	0.92	< 0.001
TS	0.93 (0.90–0.95)	2.25	0.81	< 0.001
FHL/TS	0.87 (0.82–0.91)	0.53	0.19	< 0.001
Area (mm^2^)				
FHL	0.80 (0.72–0.85)	149.35	53.88	< 0.001
TS	0.83 (0.76–0.88)	144.64	52.18	< 0.001
FHL/TS	0.83 (0.77–0.88)	1.14	0.41	< 0.001

Strength of correlation: 0.00–0.19 = very weak; 0.20–0.39 = weak; 0.40–0.59 = moderate; 0.60–0.79 = strong; 0.80–1.00 = very strong

*ICC* intraclass correlation coefficient, *%* percent, *CI* confidence interval, *MDC* minimal detectable difference, *SEM* standard error measurement, *FHL* flexor hallucis longus, *TS* triceps surae

*F test for ICC

In the tendinopathy group, there were more males and overall older patients than in the control group (*p* < 0.001). This issue was then addressed in a logistic regression model that fitted results depending on age, gender, and affected side.

As such, there was still a significant odd’s ratio = 9.56 [95% confidence interval 2.46–37.22], *p* = 0.001) for an increased ratio of diameter FHL/TS in the tendinopathy group (Table 2).

**Fig. 4:**
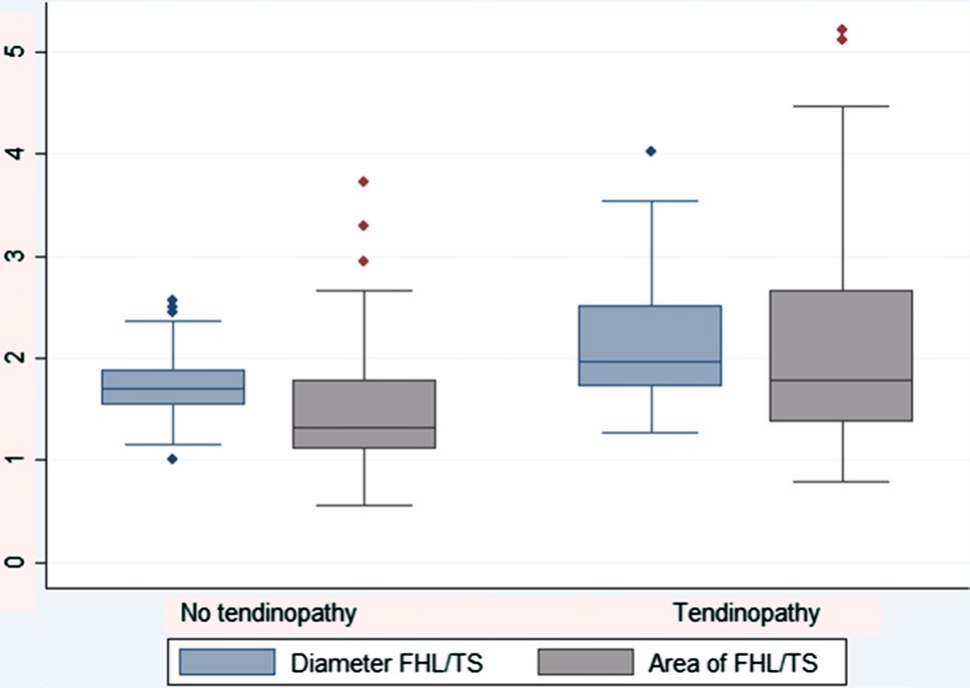
Ratios of diameter and area compared between study groups. The boxplots represent medians of the ratios of FHL/TS (blue for diameter and gray for area) (color figure online)

**Fig. 5:**
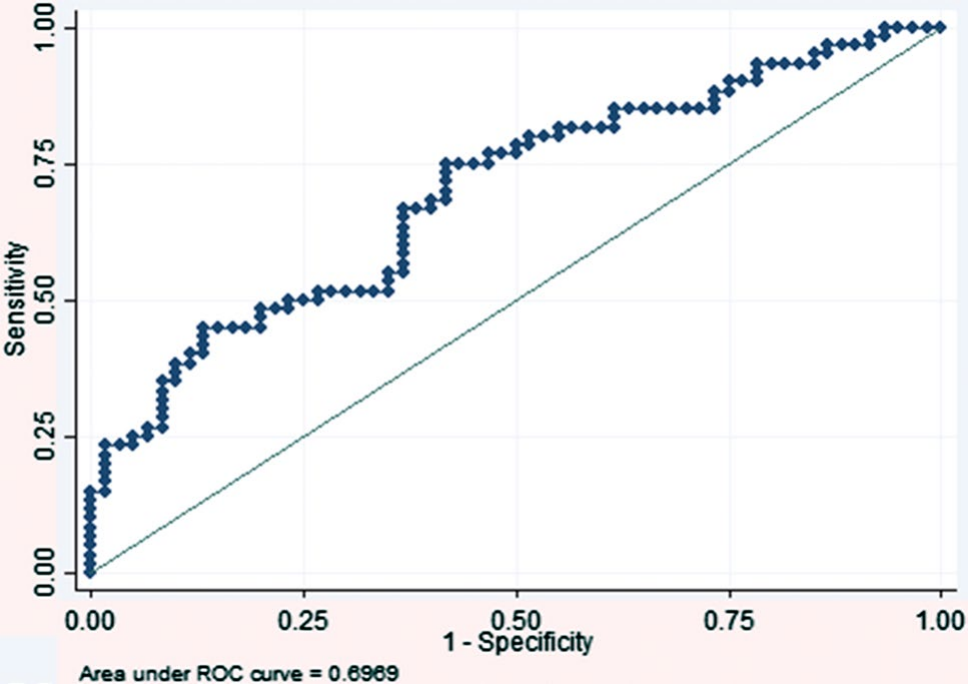
Receiver operating characteristic (ROC) curve assessing diameter ratio FHL/TS. The area under the curve (AUC) is 0.70 (95 percent [%] confidence interval [CI] 0.65–0.81). Since the lower bound of the 95% CI is > 0.50, it may be assumed that the model is suitable (i.e., superior to a random one). At a chosen cutoff point of a ratio of 2.0 and above, there was a sensitivity of 47% and specificity of 90% for concomitant signs of Achilles tendon tendinopathy

Diameter values together with its calculated quotient Dm (FHL/TS) showed less variation as demonstrated by reduced dispersion on the boxplots (Fig. 4). Therefore, it was this value that was chosen for the calculation of a potential radiographic tool. For the calculation of a suitable cutoff point for the diameter ratio Dm (FHL/TS), a receiver operating characteristic (ROC) analysis was undertaken (Fig. 5). A value of 1.6 or above was most sensitive to detect a positive correlation with concomitant tendinopathy with a sensitivity of 83% and specificity of 45%. On the other hand, a value of 2.0 or above, provided a higher specificity of 90%. The latter value was chosen as cutoff margin.

## Discussion

This is the first study reporting evidence for FHL hypertrophy that occurs in cases of severe chronic Achilles tendinopathy even before a transfer is employed. Also, the study provides a new objective radiographic tool that might help in the surgical decision-making and should be the base for future studies that will correlate it to clinical outcome.

Previous studies evaluated the reliability of ultrasound examination [21] and compared clinically symptomatic and asymptomatic Achilles tendons which found tendinopathy in 32% of the asymptomatic volunteers. [22] This demonstrates the further need for additional parameters in assessing the functionality of the Achilles tendon. Oksanen et al. found FHL hypertrophy in over 50% of patients after performing a FHL transfer. [15] Similar findings were observed and reported on postoperative MRI imaging. [15, 23, 24] We believe the hypertrophy may also occur in case of functional deterioration of the triceps surae due to chronic Achilles tendinopathy as a compensatory mechanism.

Another area of need for an additional objective tool is the indication for surgical treatment of a chronic Achilles tendinopathy which is a matter of current debate. Rahm et al. suggested to perform FHL transfer after failure of local debridement, failure of free tendon grafting in case of a tendon defect or advanced fatty infiltration of the TS [6]. A minimal defect of 50% of the tendon was determined as an indication for the need of a tendon transfer by several authors [7, 10, 11, 25] Lin et al. [26] presented an algorithm for the treatment of chronic Achilles tendinopathy based on the presence or absence of stumps on preoperative MRI and the defect gap measured intra-operatively. FHL transfer was considered when the tendon stumps did not have enough integrity [4]. Clinical results were reported to be good to excellent after FHL transfer for multiple pathologies of the Achilles tendon, including extended degenerative aberrations, partial defects, and muscle dysfunction of the TS [12, 27]. Very good ankle plantar flexion strength and an overall clinical success rate of over 70% have been reported [28–30].

As such, the management for Achilles tendinopathy experiences a variety of treatment strategies and lacks standardized criteria as it depends on subjective considerations of the caretaker [31]. The observed FHL hypertrophy in patients with signs of chronic Achilles tendinopathy provides evidence that further supports its functional synergism and as such, another argument for choosing an FHL transfer. Using the new ratio of diameter Dm (TS/FHL), an additional radiographic sign can be potentially employed in the surgical decision-making. Future clinical prospective studies should correlate this parameter to clinical and functional outcome.

Another subject of discussion may be to question the MRI as diagnostic tool for Achilles tendinopathy. MRI can be time consuming and expensive [32]. Although it was shown that it is not necessary for diagnosing acute injuries, in atraumatic cases with inconclusive clinical findings (normal or equivocal Thompson test, nonpalpable gap between tendon edges, known chronic rupture and other), it has decisive importance for diagnosis [33].

Obviously, there are several limitations that should be addressed. The design has a weakness regarding study groups that were not matched on age, BMI or comorbidities, which are possible confounders. This matter was addressed by performing a multiple regression analysis. Another issue is the cross-sectional measurement of the muscle belly at a specific location, which may be not representative of the total muscular volume. Future studies may consider potential confounders height, weight, and length of the free Achilles tendon (distance from calcaneus to tip of soleus muscle) in their analyses to account for different anatomical configurations in each individual (e.g., the left patient in Fig. 3 may have a longer Achilles tendon than the right patient, potentially leading to a smaller soleus volume 4–5 cm above the ankle joint line).

## Conclusion

In our patient cohort, flexor hallucis longus hypertrophy was observed in the Achilles tendinopathy group, probably as a compensatory mechanism. Measuring a diameter ratio Dm (FHL/TS) of 2.0 or higher on an axial MRI, may be indicative as an indirect sign of functional deterioration of the Achilles tendon.

Future studies should correlate this finding to clinical outcome, which may potentially aid in the surgical decision-making and the employment of a tendon transfer.

## Data Availability

Data were deposited safely in a local repository according to the ethical commission regulations. REDCAP software was used for storage and limited access to the principal investigators to protect patient personal information.
